# Ginger as a Potential Remedy for Periodontitis Treatment: A Review on the Present Evidence

**DOI:** 10.1002/hsr2.71052

**Published:** 2025-07-10

**Authors:** Reza Arefnehzad, Mohammad Gerayeli, Shabnam Ganjeh, Ashkan Badkoob, Ali Nassajzadeh, Reza mahmoudi, Mahsa Tebyani, Fatemeh Rezaei‐Tazangi, Amirhossein Fathi

**Affiliations:** ^1^ Student Research Committee, Fasa University of Medical Sciences Fasa Iran; ^2^ Student Research Committee, Shiraz University of Medical Sciences Shiraz Iran; ^3^ Dental Research Center Shiraz University of Medical Sciences Mashhad Iran; ^4^ Department of Anatomy School of Medicine Fasa University of Medical Sciences Fasa Iran

**Keywords:** bioactive components, ginger, inflammation, pathogenesis, periodontitis

## Abstract

**Background:**

Periodontitis is an oral inflammatory condition related to tooth‐supporting tissues (e.g., the gingiva, alveolar bone, and periodontal ligament) and affects 70%–80% of people globally. Periodontitis can be linked with some serious diseases, like cardiovascular disorders, oral cancer, and metabolic syndrome, and it has not been declared an ideal therapy with minimum side effects against it.

**Aims:**

In this narrative review, we aimed to argue documents related to periodontitis and ginger therapy, with a particular focus on involved mechanisms.

**Methods:**

This literature review was conducted on the effects of ginger administration on periodontitis treatment by screening English documents by searching related keywords, including “Periodontitis,” “Periodontal diseases,” “Periodontal disorders,” “Ginger,” “*In vitro*,” “*In vivo*,” “Experimental,” “Preclinical, “and” “Clinical” in different databases such as Google Scholar, PubMed, Scopus, Web of Science, Science Direct, and Scientific Information Databases until February 5, 2025.

**Results:**

Herbal medicine using ginger (*Zingiber officinale*) has gathered much attention in different societies due to its various pharmacological benefits, for instance, antioxidative, anti‐inflammatory, anticancer, cardioprotective, antidiabetic, chemoprotective, and immunoprotective activities. Recently, some preclinical and clinical papers have emphasized the anti‐periodontitis influences of ginger through various mechanisms, such as repressing Gram‐negative bacteria involved in disease pathogenesis, suppressing inflammatory mediators, improving periodontal parameters, and potentiating antioxidant deference system.

**Conclusion:**

Ginger would be an effective natural product that may diminish the inflammation and tissue damage caused by periodontitis.

## 1 Introduction

Periodontitis is a chronic inflammatory problem destructing protective tissues of the tooth, for instance, the gingiva, alveolar bone, and periodontal ligament (altogether named the periodontium) [1, 2]. Based on epidemiological reports, this oral problem involves 70%–80% of individuals all over the world and is related to several life‐threatening diseases, such as cardiovascular disorders, oral cancer, and metabolic syndrome. This oral condition manifests multiple signs and symptoms, like gingival bleeding, pain, flossing, receding gums, and tooth loss [3, 4, 5, 6, 7, 8]. For disease treatment, some approaches have been proposed, e.g., antibiotic therapy, root loaning, tooth scaling, and surgical methods (for the severe stage). Unfortunately, these therapeutic choices are not efficient enough and affordable [9]. Thus, the suggestion of an effective way with minimum side effects seems to be required [10]. For the time being, natural products have gained much interest in light of their effectiveness against many illnesses (such as oral disorders) and low side effects [11, 12]. In this line, one of the natural products with a herbal origin is ginger (*Zingiber officinale*) [13]. Ginger, as a spicy flavor ingredient, possesses diverse chemical constituents, comprising raw fibers, organic acid, lipids, polysaccharides, terpenes, and phenolic compounds [14, 15]. This plant has reflected its striking role in herbal medicine due to its wide pharmacological activities, like antioxidative, anti‐inflammatory, anticancer, cardioprotective, antidiabetic, chemoprotective, and immunoprotective functions [16, 17, 18, 19, 20, 21, 22]. Recently, it has been declared that ginger can be a potential candidate for periodontitis treatment [23]. Hence, we aimed to discuss the possible therapeutic capacity of this plant against this inflammatory oral disease based on the present documents.

### 1.1 Periodontitis and Pathogenic Mechanisms

**Figure 1:**
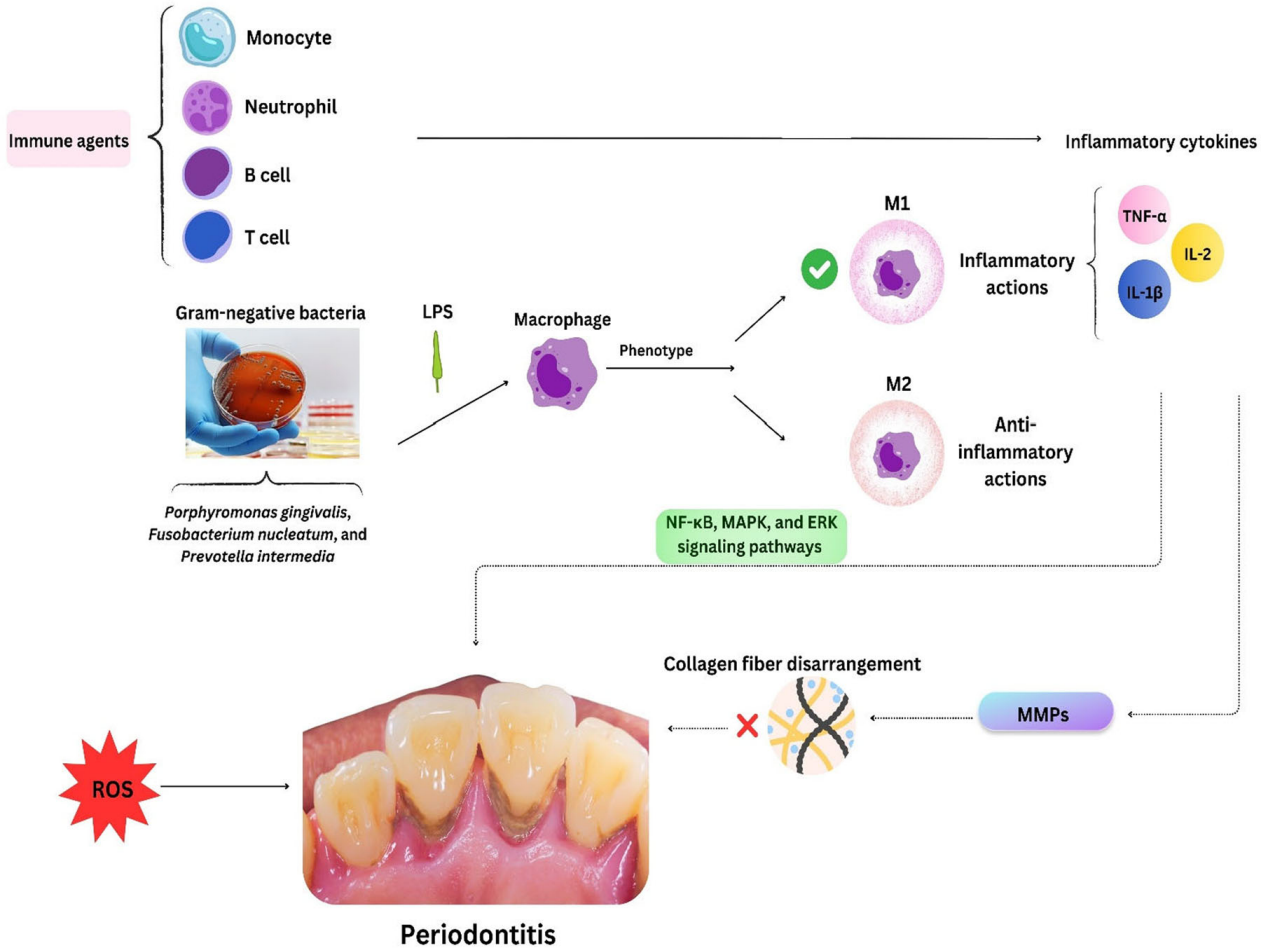
Immune, bacterial, and oxidative agents have striking role in the pathogenesis of periodontitis.

It is stated that periodontal tissue destruction, the main signature of periodontitis, occurs due to inflammatory reactions of the host immune system because of periodontal pathogens [24]. The host reactions are mainly triggered by neutrophils, B and T lymphocytes, macrophages, and monocytes (Figure 1). These immune system‐related agents can stimulate the formation of inflammatory factors, for example, cytokines, chemokines, proteolytic enzymes, and arachidonic acid metabolites, which participate in bone resorption and tissue demolition through activation of several signaling pathways, like nuclear factor kappa B (NF‐κB), mitogen‐activated protein kinase (MAPK), and extracellular‐regulated kinases (ERK) signaling pathways [25, 26, 27]. Among pro‐inflammatory cytokines, the pathogenic roles of some of them, for instance, interleukin (IL)‐1β and tumor necrosis factor‐α (TNF‐α), have been demonstrated [28, 29, 30]. Some Gram‐negative anaerobic bacteria, such as *Porphyromonas gingivalis, Fusobacterium nucleatum*, and *Prevotella intermedia*, can also activate inflammatory occurrences and finally result in tooth loss and periodontal ligament destruction [31, 32]. Indeed, these microbial pathogens activate the host macrophages and other inflammatory factors by means of their lipopolysaccharide and other virulence agents [33]. The existence of these pro‐inflammatory cytokines induces the formation of matrix metalloproteinases (MMPs) by neutrophils, junctional epithelial cells, fibroblasts, and macrophages [34, 35]. MMPs are involved in the demolition of collagen fibers, particularly in periodontal ligaments [34]. Plus, an imbalance between the production of reactive oxygen species (ROS) by phagocytic cells and their clearance in periapical lesions may lead to bone loss and periapical damage in this disease [36]. It seems that the impairment of the immune system function, antioxidant system capacity, and release of factors from dental pathogens are more involved in disease pathogenesis; however, other effective factors are recommended to be discovered in different studies.

### 1.2 Ginger Components: Their Effects on Inflammatory Mediators and Inflammatory Disorders

It has been demonstrated that ginger components, such as shogaol, gingerols, and zingerone, are chiefly responsible for the therapeutic effects of ginger, especially in inflammatory conditions [37]. 6‐shogaol is able to repress lipopolysaccharide (LPS)‐conferred formation of inflammatory factors (e.g., IL‐6 and prostaglandin E2 (PGE 2)) by suppressing nuclear translocation and phosphorylation of NF‐kB and triggering peroxisome proliferator‐activated receptor gamma (PPAR‐γ), a suppressor of NF‐kB activation, in BV2 microglia cells [38]. 6‐shogaol has also shown its potential for repressing PGE2 expression induced by IL‐1 by inhibiting the enzymatic function of PGE synthase and prostaglandin‐endoperoxide synthase 2 (PTGS2), causing reduced production of receptor activator of NF‐kB (RANKL) and so attenuation of osteoclast differentiation [39]. Similarly, 6‐gingerol, a main bioactive compound of ginger, can effectively repress the LPS‐induced expression of inducible nitric oxide synthase (iNOS), one of three important enzymes producing nitric oxide (NO) [40]. Moreover, 6‐gingerol can inhibit the phosphorylation of mammalian target of rapamycin (mTOR), Akt, and signal transducer and activator of transcription 3 (STAT3), a key signaling pathway in the inflammatory responses in macrophages [41]. Zingerone is known as a good choice for inflammatory osteolysis conferred by implanted titanium (Ti) particles. In these conditions, Zingerone is capable of inhibiting bone resorption and osteoclast differentiation by attenuating the NF‐κB signaling pathways in vitro [42]. Also, it has been pointed out that this compound is effective in decreasing the levels of high‐sensitivity C‐reactive protein (hs‐CRP), transforming growth factor‐β (TGF‐β), and IL‐6 in chronic inflammatory disorders affecting joints and bones [43]. Overall, the beneficial effects of ginger compounds comprising 6‐, 8‐, and 10‐gingerols, Zingerone, and 6‐Shogaol, on inflammatory disorders like multiple sclerosis, colitis, rheumatoid arthritis, and systemic lupus erythematosus have been approved [44, 45, 46, 47]. In conclusion, these reports suggest that components of ginger play a crucial role in anti‐inflammatory actions and therapeutic effects by modulating inflammatory factors and related agents.

### 1.3 Ginger and Its Components as Potent Candidates for Periodontitis Treatment

#### 1.3.1 Preclinical Evidence

Some published articles have emphasized the curative effects of ginger on periodontitis indirectly or directly. For example, it has been approved antibacterial influences of n‐hexane and ethanol extract of this plant on some anaerobic Gram‐negative bacteria involved in the disease pathogenesis, like *Porphyromonas gingivalis*, *Prevotella intermedia*, and *Porphyromonas endodontalis* [48]. In an in vitro and in vivo study performed by Kim et al. it was demonstrated that 6‐shogaol diminishes actin ring formation, which is necessary for bone resorption by osteoclasts and osteoclast differentiation, and suppresses osteoclast signaling pathways, such as activation of MAPK induced by RANKL, ROS production, Ca^2+^ oscillation, nuclear factor of activated T‐cells, cytoplasmic 1 (NFATc1) nuclear translocation, and nuclear factor of activated T‐cell [49]. Osteoclasts are known as specialized multinuclear cells responsible for bone resorption in vertebrates [50]. When monocyte/macrophage precursors are exposed to RANKL and macrophage‐colony stimulating factor (M‐CSF), they fuse to form functional and mature osteoclasts. The induction of these cells with RANKL triggers a signaling cascade that comprises NF‐κB and MAPK. These signaling pathways result in the activation of a key transcription factor critical for osteoclast formation, known as NFATc1 [51, 52]. On the other hand, the mobilization of intracellular calcium (Ca^2+^) via phospholipase Cγ initiates dephosphorylation mediated with calcineurin and allows NFATc1 to translocate into the nucleus. Once in the nucleus, NFATc1 binds to its own promoter, completing an “autoamplification” loop [53]. Given that unmanaged osteoclast activity has an indispensable role in bone destruction in various skeletal diseases, comprising periodontitis, anti‐resorptive approaches are developed mainly to affect the signaling pathways engaged in the function and formation of osteoclasts [54]. Kim and co‐workers also showed the decreased expression of inflammatory factors, including IL‐1β and TNF‐α, and inhibited alveolar bone resorption and osteoclastogenesis following administration of this active ingredient (10 mg/kg) in animal models of periodontitis induced by ligating around the second molar [49]. Collectively, the experimental evidence addressed that ginger can improve periodontitis probably by influencing pro‐inflammatory and bacterial agents, antioxidant systems, and osteoclastogenesis.

Interestingly, Xie et al. have suggested ginger‐derived exosome‐like nanoparticles (GELNs) in tissue damage conferred by periodontitis instead of pure ginger in vitro and in vivo [55]. GELNs are natural nanoparticles originating from ginger, consisting of a rich blend of proteins, lipids, RNA, and other molecules, with sizes varying from 50 to 500 nm. They not only convey and concentrate the active elements found in ginger but also act as a drug delivery system for information exchange, thanks to their tremendous membrane permeability [56].

*In vitro* results of the mentioned research group have revealed that GELNs can promote the multiplicity and migration of periodontal ligament fibroblasts (PDLFs), the main cells found in the periodontal membrane, and decrease ROS production by repressing the NF‐κB signaling pathway [55]. ROS serve as a double‐edged sword; at normal levels, they play a role in cell signaling, but in excess levels, they can confer oxidative stress and damage to tissues and cells directly [57, 58]. Considerable levels of ROS not only lead to impairment in the function of macrophages and neutrophils but also give rise to impairment in the regeneration of PDLFs, resulting in a decelerating improvement in periodontal disorders and attenuating the reparative ability of tissues [59]. The mentioned study provided evidence that GELNs are able to exert antioxidant effects in vitro and in vivo by potentiating the levels of catalase and total antioxidant capacity, which are usually attenuated in inflammatory conditions, and reducing malondialdehyde (MDA) levels as a lipid peroxidation index [55]. In vivo data also addressed that GELNs significantly ameliorated gum swelling and diminished periodontal pocket depth (PD). Histological assessments indicated that GELNs are superior to ginger juice in promoting the multiplicity of periodontal collagen fibers, decreasing the absorption of alveolar bone, and reducing the levels of inflammatory cells [55] (Table 1). Taken as a whole, this document accentuated the beneficial effects of GELNs on periodontitis mainly by modulating oxidative stress and inflammatory responses through suppressing of the NF‐κB signaling pathway.

**Table 1:** Preclinical studies in which the effect of ginger and its components on periodontitis has been investigated.

Ginger or its components	Effect/Mechanism	In vitro/In vivo	Reference
[10]‐gingerol and [12]‐gingerol	Antibacterial influences on some anaerobic Gram‐negative bacteria	In vitro	[47]
6‐shogaol	Diminishing actin ring formation and osteoclast differentiation and suppressing osteoclast signaling pathways	In vitro/In vivo	[49]
Ginger‐derived exosome‐like nanoparticles (GELNs)	Modulating oxidative stress and inflammatory responses through suppressing of the NF‐κB signaling pathway	In vitro	[54]

### 1.4 Clinical Evidence

There are several clinical trials showing the positive role of ginger in improving clinical indices and antioxidant agents and decreasing inflammatory factors in cases with periodontitis.

For instance, in a double‐blind, placebo‐controlled trial, these beneficial impacts on type 2 diabetes mellitus cases with chronic periodontitis were mentioned. In this clinical study, 46 patients were randomly assigned to the treatment and control groups, receiving ginger in tablet form (2 g) and placebo two times per day for 8 weeks, respectively. In this study, to evaluate the normality of distributions of variables, the Shapiro–Wilk and Kolmogorov–Smirnov tests were utilized. Also, to compare the findings between the two groups, the Independent sample t‐test was used. After ginger therapy, a remarkable decrease was found in the mean levels of TNF‐α (*p* = 0.007), hs‐CRP (*p* = 0.03), IL‐6 (*p* = 0.001), clinical attachment loss (CAL), and PD (*p* < 0.001). Also, the mean levels of glutathione peroxidase (GPx) and superoxide dismutase (SOD) in the serum of the treatment group were dramatically elevated (*p* = 0.002 and *p* = 0.001, respectively). These results highlight the striking function of ginger supplementation in the reduction of inflammatory factors and clinical indices and elevation of antioxidant markers in diabetes patients with chronic periodontitis [60]. Similarly, a double‐blind, placebo‐controlled trial has scrutinized the functionality of ginger supplement (2 g) along with nonsurgical periodontal therapy in diabetic patients with chronic periodontitis two times daily for 8 weeks [61]. Similar to the previous study, Kolmorogov–Smirnoff) and the Independent sample *t*‐test were used to statistical analyses in this investigation. The outcomes divulged that the mean levels of fasting blood glucose (FBG), MDA, glycosylated hemoglobin levels (HbA1c), PD, and CAL in the ginger group were significantly decreased (*p* < 0.05). Also, the mean levels of high‐density lipoprotein (HDL) in the serum of subjects who consumed ginger were considerably elevated (*p* < 0.05). Moreover, between the treatment and place groups, there were considerable differences in the average changes of HDL (3.95 ± 8.54 vs. −0.76 ± 5.04; *p*  = 0.03), HbA1c (−0.75 ± 1.17 vs. −0.16 ± 0.44; *p* = 0.04), PD (−0.52 ± 0.51 vs. −0.19 ±  0.51; *p* = 0.04), and CAL (−0.57 ± 0.50 vs. −0.14 ± 0.35; *p* = 0.003). These data suggested that ginger therapy accompanied by nonsurgical periodontal therapy can be a suitable option for regulating lipid, antioxidant, glycemic and periodontal factors in diabetic subjects with chronic periodontitis [61].

**Figure 2:**
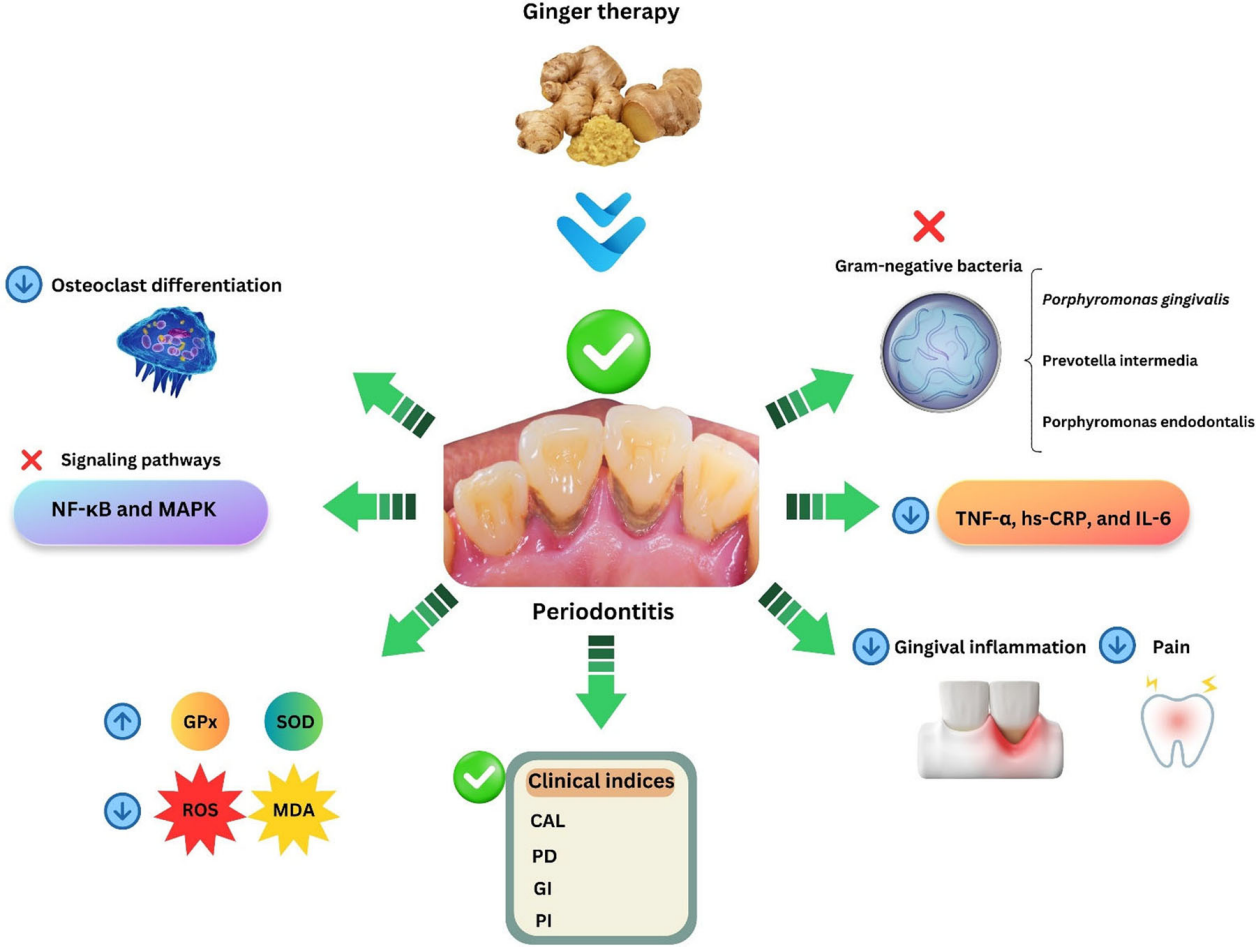
Ginger can be effective against periodontitis by affecting pathogenic mechanisms of this disease.

Recently, Alshibani et al. assessed the effects of the administration of ginger tablets (400 mg) in patients with periodontitis (Group 1) compared with cases treated with nonsteroidal anti‐inflammatory drugs (NSAIDs) (400 mg) (Group 2) [62]. Statistically, periodontal parameters and self‐rated pain scores were compared at the initiation and follow‐up by one‐way ANOVA test with Bonferroni post‐hoc adjustment. In this project, all participating subjects were diagnosed with stage II, grade B periodontitis. They found that in these two groups, there was a considerable diminish in PD (*p* < 0.01), gingival index (GI) (*p* < 0.01), and plaque index (PI) (*p* < 0.01) in the follow‐up duration (at 7, 14 and 21 days) in comparison with the baseline status. Overall, the authors displayed that this herbal product, similar to NSAIDs, ameliorates postoperative pain and periodontal landmarks like PD, GI, and PI (Figure 2) [62].

In another project, 10 subjects with chronic generalized periodontitis were chosen for appraising the effectiveness of capsule form of ginger powder (400 mg) compared with ibuprofen (400 mg), as a nonsteroidal anti‐inflammatory drug, on gingival inflammation and pain after open flap debridement in a randomized cross‐over clinical investigation. For statistical analyses, the Mann–Whitney U test was utilized to evaluate the statistical significance of the difference in mean pain scores postoperatively between ginger and ibuprofen. It was also applied to assess the difference on average Modified Gingival Index (MGI) scores for both groups. In this study, data related to gingival inflammation and pain score were evaluated using MGI and the Visual Analog Scale (VAS), respectively. The findings showed that there were no significant differences in terms of the MGI index between the two groups. This trend was also observed in the pain score between the two groups at every 1‐h interval. However, the average value of the pain score in the ginger therapy group (2.4 ± 1.07) was higher than the ibuprofen therapy group (1.5 ± 0.97), revealing a significant difference statistically. Overall, this scientific project indicated that ginger could be effective, similar to ibuprofen, in the management of gingival inflammation and pain after periodontal surgery [63] (Table 2).

**Table 2:** Some of clinical trials for application of ginger and its components for periodontitis treatment.

Duration of treatment	Dose	Total no. of patients	Findings	Reference
8 weeks	2 g	46	Decreasing the mean levels of TNF‐α, hs‐C‐reactive protein and improving clinical attachment loss and pocket depth	[59]
8 weeks	2 g	50	Decreasing the mean levels of fasting blood glucose, malondialdehyde, and glycosylated hemoglobin levels (HbA1c) and improving clinical attachment loss and pocket depth	[60]
7, 14, and 21 days	400 mg	30	Diminishing pocket depth, gingival index, and plaque index	[61]
3 days	400 mg	10	Ginger could be effective similar ibuprofen in the management of gingival inflammation and pain after periodontal surgery	[62]

### 1.5 Ginger: Prospects and Challenges

Ginger is gaining popularity due to increased public health awareness concerning its high nutritional values and health benefits [64]. According to the preclinical and clinical investigations mentioned above, one of the important therapeutic effects of ginger is related to its potential to ameliorate periodontitis. The current evidence shows that ginger, thanks to its different components (e.g., shogaol, gingerols, and zingerone), can be effective in periodontitis by repressing factors or signaling pathways associated with inflammatory reactions and improving periodontal tissue repair. Experimental data emphasize that ginger and its components have a good capacity to dwindle bone absorption, attenuate osteoclast differentiation, and ROS formation, and act against anaerobic Gram‐negative bacteria involved in the disease pathogenesis. Preclinical evidence also supports these results; it has been implicated that the consumption of a ginger supplement not only regulates the levels of inflammatory factors and antioxidant defense system but also promotes the periodontal clinical outcomes as well as ameliorate gingival inflammation and pain score in patients with periodontitis. Interestingly, the anti‐peritonitis influences of ginger can be similar to some NSAIDs, like ibuprofen. However, a clinical study showed that subjects who consume ginger three times daily at a dosage of 400 mg per dose orally for 2 weeks can experience heartburn and mild diarrhea. Additionally, it was mentioned that an escalating intake of more than 6 grams of ginger led to significant gastric irritation [65]. On the other hand, from the pharmacological viewpoint, there are some challenges to the extensive use of ginger in clinical practice, such as poor bioavailability and stability [55]. Therefore, finding effective ways to harness the therapeutic capacity of this natural agent has become a critical subject. These days, harnessing drug delivery systems, such as polymers, liposomes, and other nanoparticles, have shown promise in the treatment of different diseases. Drug delivery systems are known as devices or formulations that allow the entrance of therapeutic agents into the body, leading to promoting their safety and efficacy by controlling the time, rate, and site of drug release into the human body [66]. Thus, more research to develop and design nano‐based formulations of ginger for effective treatment of periodontitis is strongly proposed.

## 2 Conclusion

In summary, experimental and clinical reports of recent documents have proposed herbal therapy using ginger as a suitable candidate for treating periodontitis. The current preclinical studies indicated that ginger as well as its compounds, especially 6‐Shogaol, have a striking role in suppressing Gram‐negative bacterial involved in disease incidence (e.g., *Porphyromonas gingivalis*, *Fusobacterium nucleatum*, and *Prevotella intermedia*), blocking the formation of inflammatory agents (e.g., prostaglandins, and leukotrienes), and inhibiting osteoclast‐related signaling pathways (like MAPK and NF‐κB signaling pathways), ROS production, Ca^2+^ oscillation, NFATc1, and nuclear factor of activated T‐cell as well as osteoclast differentiation, leading to attenuating alveolar bone resorption. Also, the elimination of MMPs has been usually considered as a subsidiary therapeutic strategy in periodontal diseases. Ginger has been shown to remarkably diminish MMP‐2 and MMP‐9, as well as MMP‐1 and MMP‐8, the two main collagenases, expression in Human gingival fibroblasts (HGFs) when compared with lipopolysaccharides‐stimulated. The clinical evidence has supported these outcomes and showed that this herbal product is able to decrease the levels of inflammatory mediators (IL‐6, IL‐1β, TNF‐α, and hs‐CRP), and improve clinical indices (e.g., CAL, PD, GI, and PI).

It can be concluded that ginger would be an effective natural product that may help mitigate inflammation and tissue damage characteristics of periodontitis. Furthermore, given its minor adverse effects on cell viability as demonstrated by the results of cytotoxicity assays, it is expected to be safe and have minimal adverse effects on tissues. However, more research are needed to validate these results.

## Author Contributions

**Reza Arefnehzad:** validation, formal analysis, writing – original draft. **Mohammad Gerayeli:** writing – original draft. **Shabnam Ganjeh:** writing – original draft, software. **Ashkan Badkoob:** software, writing – original draft, formal analysis. **Ali Nassajzadeh:** investigation, writing – original draft, formal analysis. **Reza mahmoudi:** investigation, validation, software, writing – original draft. **Mahsa Tebyani:** writing – original draft, visualization. **Fatemeh Rezaei‐Tazangi:** data curation, supervision, writing – review and editing. **Amirhossein Fathi:** project administration, resources.

## Ethics Statement

Ethical issues (including plagiarism, data fabrication, double publication) have been completely observed by the authors.

## Conflicts of Interest

The authors declare no conflicts of interest.

## 1 Transparency Statement

The lead authors Fatemeh Rezaei‐Tazangi and Amirhossein Fathi affirm that this manuscript is an honest, accurate, and transparent account of the study being reported; that no important aspects of the study have been omitted; and that any discrepancies from the study as planned (and, if relevant, registered) have been explained.

## Data Availability

Data available on request from the authors.
